# Supplementary material for: Combined Treatment with MEK and mTOR Inhibitors is Effective in In Vitro and In Vivo Models of Hepatocellular Carcinoma
Source: Cancers (Basel). 2019 Jul 3;11(7):930. doi: 10.3390/cancers11070930 (PMC6679026; doi:10.3390/cancers11070930)
Supplement: Supplementary file 1 [file cancers-11-00930-s001.pdf]

# Supplementary Material: Combined Treatment with MEK and mTOR Inhibitors is Effective in In Vitro and In Vivo Models of Hepatocellular Carcinoma

**Xianqiong Liu, Junjie Hu, Xinhua Song, Kirsten Utpatel, Yi Zhang, Pan Wang, Xinjun Lu, Jie Zhang, Meng Xu, Tao Su, Li Che, Matthias Evert, Diego F. Calvisi and Xin Chen**

**Table 1.** Immunohistochemistry and Western blotting antibody information.

| Antibody | Company | Catalog number | Hosts of antibodies | Species Reactivity | Class | Dilution | Method |
|---|---|---|---|---|---|---|---|
| Ki67 | Thermo Scientific | MA5-14520 | Rabbit | H | Monoclonal | 1:100 | IHC |
| Cleaved-caspase-3 | Cell Signaling Technology | 9664 | Rabbit | H, M, R, Mk | Monoclonal | 1:500 | IHC |
| AKT | Cell Signaling Technology | 9272 | Rabbit | H, M, R, Hm, Mk, C, Dm, B, Dg, Pg, GP | Polyclonal | 1:1000 | WB |
| Phospho-AKT$^{S473}$ | Cell Signaling Technology | 3787 | Rabbit | H, M | Monoclonal | 1:1000 | WB |
| Phospho-mTOR | Cell Signaling Technology | 2971 | Rabbit | H, M, R, Mk | Polyclonal | 1:1000 | WB |
| T-mTOR | Cell Signaling Technology | 2983 | Rabbit | H, M, R, Mk | Monoclonal | 1:1000 | WB |
| ERK | Cell Signaling Technology | 9102 | Rabbit | H, M, R, Hm, Mk, Mi, Z, B, Pg, Sc | Polyclonal | 1:1000 | WB |
| Phospho-ERK | Cell Signaling Technology | 4370 | Rabbit | H, M, R, Hm, Mk, Mi, Dm, Z, B, Dg, Pg, Sc | Monoclonal | 1:1000 | WB |
| S6 | Cell Signaling Technology | 2217 | Rabbit | H, M, R, Mk | Monoclonal | 1:1000 | WB |
| Phospho-RPS6 | Cell Signaling Technology | 4858 | Rabbit | H, M, R, Mk, Mi, Sc | Monoclonal | 1:2000 | WB |
| Phospho-4E-BP1 | Cell Signaling Technology | 2855 | Rabbit | H, M, R, Mk, Dm | Monoclonal | 1:1000 | WB |
| Phospho-4E-BP1(S65) | Cell Signaling Technology | 9451 | Rabbit | H, M, R, Mk | Polyclonal | 1:1000 | WB |

| | | | | | | | |
|---|---|---|---|---|---|---|---|
| 4E-BP1 | Cell Signaling Technology | 9644 | Rabbit | H, M, R, Mk | Monoclonal | 1:1000 | WB |
| Phospho-elF4E | Cell Signaling Technology | 9741 | Rabbit | H, M, R, Mk | Polyclonal | 1:1000 | WB |
| c-Met | invitrogen | 71-8000 | Rabbit | H, M | Polyclonal | 1:400 | WB |
| p-Met | Cell Signaling Technology | 3129 | Rabbit | H, M, R | Monoclonal | 1:1000 | WB |
| Survivin | Cell Signaling Technology | 2808 | Rabbit | H, M, R | Monoclonal | 1:1000 | WB |
| PCNA | Cell Signaling Technology | 2586 | Mouse | H, M, R, Mk, B, Pg | Monoclonal | 1:2000 | WB |
| Cyclin A | Santa Cruz Biotechnology | SC-751 | Rabbit | H, M, R | Polyclonal | 1:200 | WB |
| Cyclin B1 | Santa Cruz Biotechnology | SC-245 | Mouse | H, M, R | Monoclonal | 1:750 | WB |
| Cyclin D1 | Cell Signaling Technology | 2978 | Rabbit | H, M, R | Monoclonal | 1:10000 | WB |
| Cyclin E | Biolegend | 630701 | Rabbit | H, M, R | Monoclonal | 1:200 | WB |
| Cleaved-caspase3 | Cell Signaling Technology | 9664 | Rabbit | H, M, R, Mk | Monoclonal | 1:750 | WB |
| Cleaved-caspase7 | Cell Signaling Technology | 8438 | Rabbit | H, M, R | Monoclonal | 1:750 | WB |
| Bcl-2 | Cell Signaling Technology | 3498 | Rabbit | H, M | Monoclonal | 1:1000 | WB |
| Bcl-xI | Cell Signaling Technology | 2764 | Rabbit | H, M, R, Mk | Monoclonal | 1:1000 | WB |
| Bim | Cell Signaling Technology | 2933 | Rabbit | H, M, R | Monoclonal | 1:1000 | WB |
| Mcl-1 | Cell Signaling Technology | 94296 | Rabbit | H, M, R | Monoclonal | 1:1000 | WB |
| LC-3(A/B) | Cell Signaling Technology | 12741 | Rabbit | H, M, R | Monoclonal | 1:1000 | WB |
| β-ACTIN | Sigma-Aldrich | A5441 | Mouse | H, M, R | Monoclonal | 1:4500 | WB |
| GAPDH(D16H11) | Cell Signaling Technology | 5174 | Rabbit | H, M, R, Mk | Monoclonal | 1:10000 | WB |

Abbreviations: H, Human; M, Mouse; R, Rat; Ha, Hamster; Mk, Monkey; C, Chicken; Dm, D. melanogaster; B, Bovine; Dg, Dog; Pg, Pig; GP, Guinea Pig.
